# Identifying causal genes for migraine by integrating the proteome and transcriptome

**DOI:** 10.1186/s10194-023-01649-3

**Published:** 2023-08-17

**Authors:** Shuang-jie Li, Jing-jing Shi, Cheng-yuan Mao, Chan Zhang, Ya-fang Xu, Yu Fan, Zheng-wei Hu, Wen-kai Yu, Xiao-yan Hao, Meng-jie Li, Jia-di Li, Dong-rui Ma, Meng-nan Guo, Chun-yan Zuo, Yuan-yuan Liang, Yu-ming Xu, Jun Wu, Shi-lei Sun, Yong-gang Wang, Chang-he Shi

**Affiliations:** 1 Department of Neurology, The First Affiliated Hospital of Zhengzhou University, Zhengzhou University, Zhengzhou, 450000 Henan China; 2 Henan Key Laboratory of Cerebrovascular Diseases, The First Affiliated Hospital of Zhengzhou University, Zhengzhou University, Zhengzhou, 450000 Henan China; 3 Institute of Neuroscience, Zhengzhou University, Zhengzhou, 450000 Henan China; 4 Headache Center, Department of Neurology, Beijing Tiantan Hospital, Capital Medical University, No.119 South Fourth Ring West Road, Fengtai District, Beijing, 100070 China

**Keywords:** Migraine, Transcriptome-wide association study, Proteome-wide association studies, Fine-mapping

## Abstract

**Background:**

While previous genome-wide association studies (GWAS) have identified multiple risk variants for migraine, there is a lack of evidence about how these variants contribute to the development of migraine. We employed an integrative pipeline to efficiently transform genetic associations to identify causal genes for migraine.

**Methods:**

We conducted a proteome-wide association study (PWAS) by combining data from the migraine GWAS data with proteomic data from the human brain and plasma to identify proteins that may play a role in the risk of developing migraine. We also combined data from GWAS of migraine with a novel joint-tissue imputation (JTI) prediction model of 17 migraine-related human tissues to conduct transcriptome-wide association studies (TWAS) together with the fine mapping method FOCUS to identify disease-associated genes.

**Results:**

We identified 13 genes in the human brain and plasma proteome that modulate migraine risk by regulating protein abundance. In addition, 62 associated genes not reported in previous migraine TWAS studies were identified by our analysis of migraine using TWAS and fine mapping. Five genes including *ICA1L*, *TREX1*, *STAT6*, *UFL1*, and *B3GNT8* showed significant associations with migraine at both the proteome and transcriptome, these genes are mainly expressed in ependymal cells, neurons, and glial cells, and are potential target genes for prevention of neuronal signaling and inflammatory responses in the pathogenesis of migraine.

**Conclusions:**

Our proteomic and transcriptome findings have identified disease-associated genes that may give new insights into the pathogenesis and potential therapeutic targets for migraine.

**Supplementary Information:**

The online version contains supplementary material available at 10.1186/s10194-023-01649-3.

## Background

Migraine is one of the most disabling diseases globally [1], characterized by recurrent, severe headaches often accompanied by a range of associated symptoms such as sensitivity to light, sound, and smell, nausea, and vomiting [2, 3]. It is a genetically complex neurological disorder, significantly influenced by genetic factors with a heritability estimated at up to 57% [4].

Several genome-wide association studies (GWAS) have been conducted to identify potential genetic risk factors for migraine. Gormley et al. applied meta-analysis to migraine GWAS to identify genomic loci [5]. Subsequent enrichment analysis revealed an association with vascular and smooth muscle tissue, supporting the vascular theory of migraine [5]. In a study involving 873,341 participants, including 102,084 cases and 771,257 controls, 123 migraine-associated loci were identified. It was found that these genes were predominantly enriched in the central nervous system and the vascular system. Transcriptome-wide association study (TWAS) is a method used to investigate the correlation between the transcriptome and each gene locus [6]. Similarly, proteome-wide association studies (PWAS) combine GWAS data with proteomic data to identify candidate genes associated with a given trait [7].

In this study, we used migraine GWAS data in conjunction with the human brain and plasma proteome for PWAS [7]. We also employed the joint-tissue imputation (JTI) prediction model across 17 tissues in migraine GWAS for TWAS [6, 8], followed by fine mapping (FOCUS) [9, 10], to identify risk genes associated with the proteome and transcriptome of migraine. Our findings provide insight into the potential biological mechanisms by which these genes contribute to the development of migraine.

## Materials and methods

### Migraine GWAS data

**Fig. 1:**
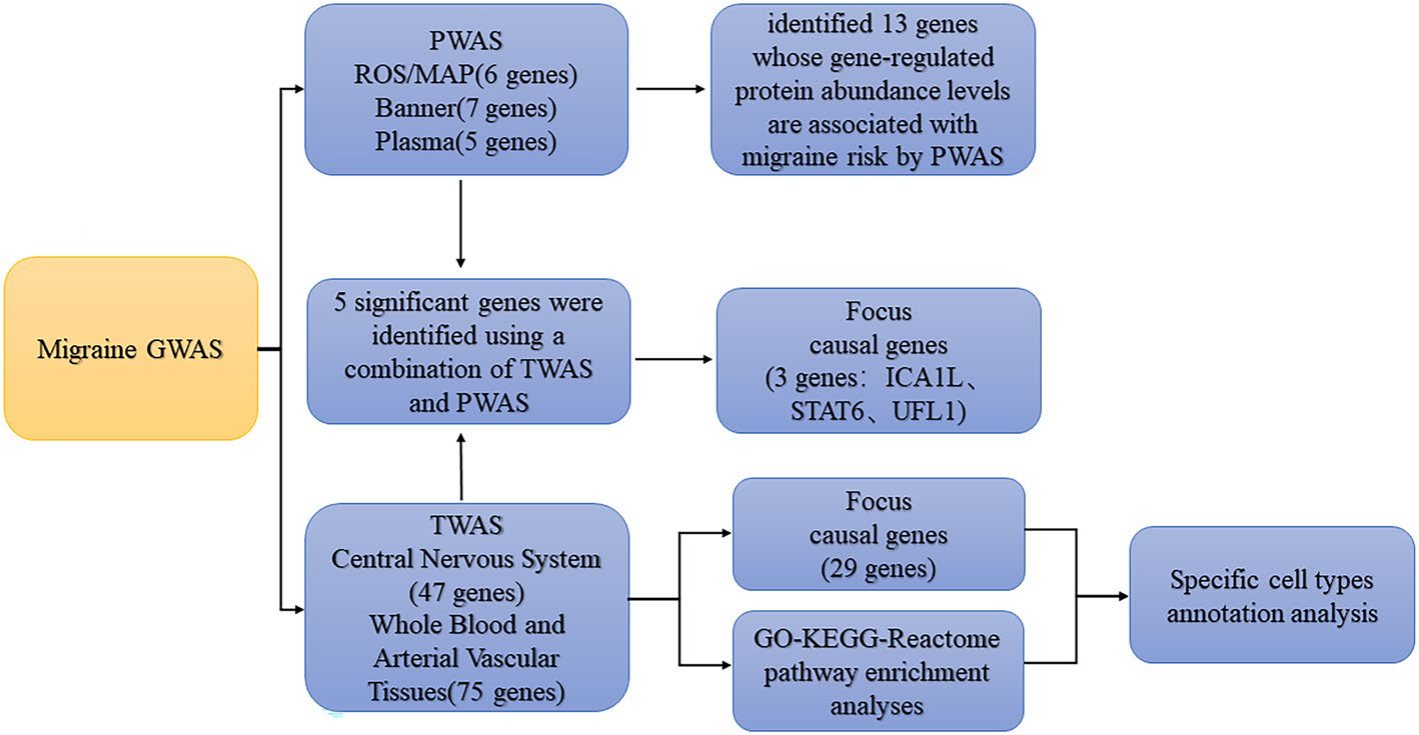
The workflow of the study. PWAS, proteome-wide association study; TWAS, transcriptome-wide association study; FOCUS, Fine-mapping of causal gene sets

In this study, we utilized the genome-wide summary statistics from the International Headache Genetics Consortium (IHGC) to identify risk loci for migraine. The IHGC dataset consists of 48,975 cases of migraine and 540,381 controls, all of European ethnicity. This large sample size allows for robust analysis and increases the statistical power to detect significant associations. The GWAS data underwent rigorous quality control (QC) measures, including checks for genotyping errors, minor allele frequency, and Hardy–Weinberg equilibrium. The final dataset used for analysis included a specific number of single nucleotide polymorphisms (SNPs) that passed these QC measures. The exact number of SNPs used will be provided upon completion of the QC process [11]. Figure 1 summarizes the various analytical steps performed on the GWAS dataset.

### Proteomic data

In this study, we profiled human brain proteomes from the dorsolateral prefrontal cortex (dPFC) of post-mortem brain samples donated by participants of European descent. These samples were sourced from the Religious Orders Study and Rush Memory and Aging Project (ROS/MAP), and the Banner Sun Health Research Institute (Banner). The ROS/MAP dataset includes proteomic and genetic data from 376 subjects, with 8,356 proteins passing quality control for protein quantitative trait locus (pQTL) analysis. The Banner dataset includes data from 152 subjects, with 8,168 proteins passing quality control for pQTL analysis [12, 13]. We also utilized a plasma protein dataset consisting of 4,657 proteins from 7,213 European-Americans [14]. All of these datasets have undergone rigorous quality control to ensure accuracy and reliability of the data [15]. The ROS/MAP and Banner datasets include samples from both old and young patients, as well as controls. However, it's important to note that these datasets do not specifically indicate whether the samples were from migraine cases or what percentage of the subjects had migraine. This is a limitation of these datasets, and it could potentially impact our understanding of the relationship between genetic variants and migraine relevance at the protein and RNA level.

### Proteome-wide association study

To perform the proteome-wide association study (PWAS), we adopted the functional summary-based imputation (FUSION) method to combine the genetic effect of migraine (GWAS Z-score) with protein weights. FUSION is a computational method designed to integrate functional genomic data with GWAS summary statistics, thereby enhancing the imputation of the GWAS summary statistics [16]. Initially, we employed a linkage disequilibrium (LD) reference panel downloaded from FUSION website. The purpose of this was to mitigate the influence of LD on the estimated test statistics. Following this, we estimated the SNP-based heritability for each gene, utilizing both proteomic and genetic data. We used FUSION to compute the effect of SNPs with significant heritability (*p* value < 0.01) on protein abundance using multiple predictive models (top1, blup, lasso, enet, and bslmm). The model that yielded the most predictive results was subsequently used for the protein weights. We used FUSION to combine the genetic effect of migraine (migraine GWAS Z-score) with the protein weights by calculating the linear sum of Z-score × weight for the independent SNPs at the locus to perform the PWAS of migraine. The results were adjusted using the Bonferroni multiple testing correction (pBonferroni < 0.05/total number of genes included in the analysis in each data). This approach allowed us to identify proteins that may be involved in the risk of developing migraine and to gain a deeper understanding of the underlying mechanisms of the disorder [7, 15].

### Joint-tissue imputation (JTI) models

Joint-tissue imputation (JTI) models are pre-training models obtained on the basis of multi-tissue transcriptome data (GTEx v8), considering shared genetic effects of regulation between different tissues and unique genetic regulation in the target tissue[8]. Here, we obtained prediction models for 17 tissues, including 13 brain tissues (amygdala, anterior cingulate cortex BA24, caudate basal ganglia, cerebellar hemispheres, cerebellum, cerebral cortex, anterior cerebral cortex BA9, hippocampus, hypothalamus, volar nucleus basal ganglia, Choroidal nucleus basalis ganglia, cerebral spinal cord cervical c-1, brain substantia nigra), whole blood, and 3 vascular tissues (aorta arteries, tibial arteries, and coronary arteries). These tissues were chosen due to their relevance to the pathophysiology and LDSC-SEG results of migraine [11, 17, 18]. The JTI method allowed us to identify genetic variants associated with migraine in multiple tissues, providing insight into the complex genetic basis of this neurological disorder.

### Transcriptome-wide association study

S-PrediXcan is an approach used to predict gene expression levels based on genetic data, specifically single nucleotide polymorphisms (SNPs), and a reference panel of gene expression data [19, 20]. It estimates gene expression weights by training a linear prediction model using a reference sample that includes both gene expression and SNP genotype data. In our application of S-PrediXcan, we used migraine GWAS summary statistics as the study set. We utilized expression weights for 17 tissues with S-PrediXcan expression weights from the JTI model, and LD information from the 1000 Genomes Project Phase 3. To address the issue of multiple testing, we employed Bonferroni multiple testing correction to adjust the significance threshold (*p* value). Genes with *p* value lower than the Bonferroni-corrected threshold were considered potentially significant in relation to migraine.

### TWAS fine mapping

To identify relationships between different characteristics within specific genetic regions, we used a method known as TWAS fine mapping, specifically employing the FOCUS method [9, 10]. This method helps us estimate the likelihood that a particular genetic feature is involved in causing the trait of interest. It does this by combining data from GWAS, which look at the entire genome, with expression quantitative trait loci (eQTL) analysis, which examines how genetic variations influence gene expression [10]. A key metric in this process is the posterior inclusion probability (PIP), which is the estimated probability that a particular genetic feature is involved in the trait of interest. In statistical terms, PIP is the marginal posterior probability that a variable (in this case, a genetic feature) should be included in the model. If the PIP is greater than 0.9, this suggests that we can be 90% confident that the genetic feature plays a role in the development and manifestation of the trait. In simpler terms, a high PIP indicates a strong likelihood that the gene is involved in the trait studying. The FOCUS method has been shown to improve the precision of identifying these causal genes and is more sensitive compared to other methods. This makes it a powerful tool for identifying genes associated with diseases.

### TWAS-based gene set enrichment analysis

Following the identification of risk genes through TWAS analysis, we categorized them based on their Z-score. Genes with Z-score greater than 0 were classified as risk genes, suggesting their potential role in increasing the likelihood of developing migraines. Conversely, genes with Z-score less than 0 were considered protective factors against migraines. This categorization was conducted for genes obtained from TWAS analyses of the central nervous system (CNS), whole blood, and vascular tissues. To further investigate the roles of these risk and protective genes, we employed several analytical tools. We used the Enrichr online tool to conduct a Gene Ontology (GO) analysis, a Kyoto Encyclopedia of Genes and Genomes (KEGG) pathway analysis, and a Reactome database analysis [21]. These analyses aimed to explore the specific pathways and processes associated with the identified genes. Finally, to visualize the network of these pathways, we utilized the Metascape online tool [22]. By using these tools, we aim to provide a more comprehensive understanding of the genetic underpinnings of migraines.

### Cell type specificity analysis

CoExp Web is an online tool that allows for the annotation of genes using co-expression networks based on brain transcriptomic data or transcriptomic data from other tissues [23]. We utilized this tool in our study to identify specific cell types that may be involved in the pathogenesis of migraine by using transcriptomic data from the brain and other tissues [24]. This approach allows us to gain a deeper understanding of the cell types that play a key role in the development and manifestation of the disorder.

## Results

### PWAS of migraine

**Fig. 2:**
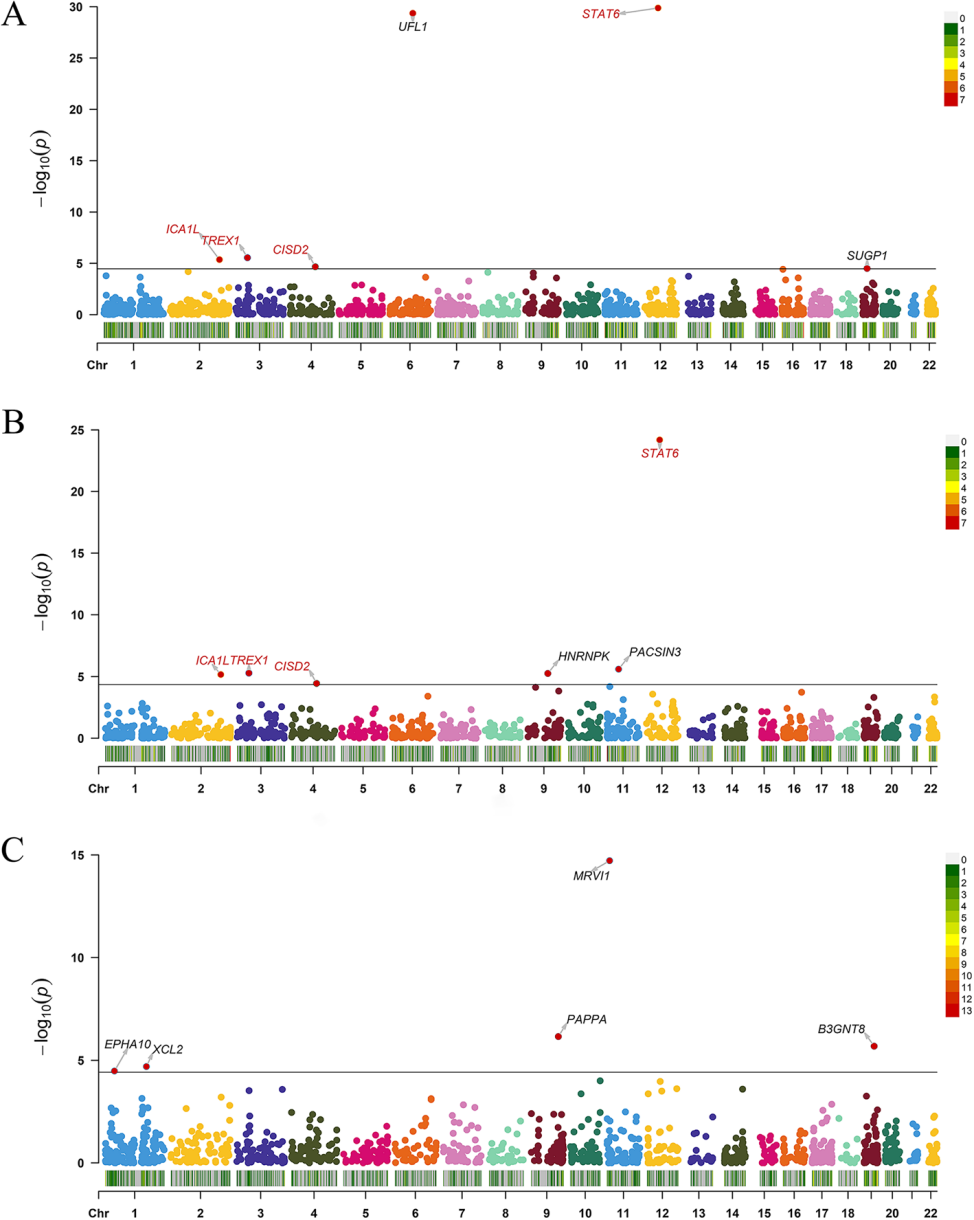
Manhattan plots for the migraine PWASs in the human brain and plasma proteomes. Manhattan plot for the PWAS integrating the migraine GWAS with the ROSMAP proteomes (*n* = 376) (A), Banner proteomes (*n* = 152) (B), and plasma proteomes (*n* = 152) (C). Each dot on the x-axis represents a gene, and the association strength on the y-axis represents the -log10(p) of PWAS. Proteome-wide significance level in the ROSMAP dataset was set at *p* < 4.363 × 10–5(adjusted by Bonferroni multiple testing correction method). Proteome-wide significance level was set at *p* < 4.41 × 10–5 (adjusted by the method of multiple testing correction is the Bonferroni adjustment.) for the Banner dataset. Proteome-wide significance level in the ROSMAP dataset was set at p < 3.71 × 10–5(adjusted by Bonferroni multiple testing correction method). Proteome-wide significant genes (*ICA1L*, *TREX1*, *CISD2*, and *STAT6*) in both brain proteomes are shown in red. Chr, chromosome

In our study, we identified six genes (*CISD2*, *ICA1L*, *STAT6*, *SUGP1*, *TREX1*, and *UFL1*) through PWAS approach. This approach involved integrating proteomics data from the ROS/MAP with migraine GWAS data (Fig. 2A and Additional file 1: Table S3). The PWAS approach works by combining genetic data with protein abundance data to identify genes that may influence the risk of migraine by regulating protein abundance in the brain. The significance of these genes was determined using a Bonferroni multiple testing correction, with a stringent *p* value threshold set at 4.363E-5. We also identified two additional genes, *HNRNPK* and *PACSIN3*, in the PWAS by integrating proteomics data from the Banner Sun Health Research Institute with migraine GWAS data (Fig. 2B and Additional file 1: Table S4). Furthermore, we integrated the plasma proteomic dataset with migraine GWAS data for another PWAS, identifying five more genes (*MRVI1*, *PAPPA*, *B3GNT8*, *XCL2*, and *EPHA10*) as potential risk genes (Fig. 2C and Additional file 1: Table S5). These genes may influence the risk of migraine by regulating plasma protein abundance. In total, we identified eight candidate risk genes for migraine using brain pQTL, and five candidate risk genes using plasma pQTL. pQTLs are genetic locations that are associated with variations in protein levels. And, we did not observe a clear overlap or trend between the significant proteins identified by brain-based pQTL and those identified by plasma-based pQTL. *CISD2*, *ICA1L*, *STAT6*, and *TREX1* all demonstrated significance at the Bonferroni multiple testing corrected *p* value threshold between two brain proteomics data (Table 1).

**Table 1:** Candidate genes in proteomes associated with migraine

Gene	Chr	PWAS						TWAS significant
		ROSMAP		Banner		Plasma		
		*Z-score*	*p-value*_*a*_	*Z-score*	*p-value*_*b*_	*Z-score*	*p-value*_*c*_	
*XCL2*	1	-	-	-	-	4.2627	2.02E-05	-
*EPHA10*	1	-	-	-	-	-4.1497	3.33E-05	-
***ICA1L***	2	-4.59488	4.33E-06	-4.5015	6.75E-06	-	-	Suggestive
***TREX1***	3	-4.6832	2.82E-06	-4.5604	5.11E-06	-	-	Suggestive
***CISD2***	4	4.2477	2.16E-05	4.1343	3.56E-05	-	-	-
*UFL1*	6	11.3956	4.4E-30	-	-	-	-	Suggestive
*HNRNPK*	9	-	-	4.5444	5.51E-06	-	-	-
*PAPPA*	9	-	-	-	-	4.9603	7.04E-07	-
*PACSIN3*	11	-	-	-4.7089	2.49E-06	-	-	-
*MRVI1*	11	-	-	-	-	-7.94798	1.90E-15	-
***STAT6***	12	-11.499	1.33E-30	-10.3081	6.48E-25	-	-	Suggestive
*SUGP1*	19	-4.1653	3.11E-05	-	-	-	-	-
*B3GNT8*	19	-	-	-	-	4.74602	2.07E-06	Suggestive

*PWAS* proteome-wide association study

^a^Proteome-wide significance level in the ROSMAP dataset was set at p < 4.363E-5 (adjusted by Bonferroni multiple testing correction method)

^b^Proteome-wide significance level in the Banner dataset was set at *p* < 4.41E-5 (adjusted by Bonferroni multiple testing correction method)

^c^Proteome-wide significance level in the Plasma dataset was set at *p* < 3.71E-5 (adjusted by Bonferroni multiple testing correction method)

The genes in bold are the ones that are significant in both the proteome-wide and transcriptome-wide levels

### TWAS analysis

In the TWAS analysis using the JTI reference transcriptome interpolation model, we identified 95 genes associated with migraine (Additional file 1: Tables S6). Among these, 47 were found in CNS tissues, 75 were found in whole blood and vascular tissues, with 27 overlapping (Additional file 1: Tables S7-8, Additional file 2: Figures S1 and Table S1). Of these, 33 have been reported in previous studies, while the remaining 62 genes are newly identified risk genes (Additional file 1: Tables S9-10). Five genes, including *ICA1L*, *TREX1*, *STAT6*, *UFL1*, and *B3GNT8*, showed significant correlation with migraine in both the proteome and transcriptome (Table 1). In this study, FOCUS identified 33 genes with a strong causal association with migraine, of which 29 overlapped with the results identified by TWAS (Additional file 1: Table S11, Additional file 2: Figure S2). Of these 29 genes identified by both TWAS and FOCUS analyses, 10 were novel genes not previously reported in migraine-related GWAS studies (Additional file 1: Table S12; Table 2).

**Table 2:** Ten identified gene has not been reported to be associated with migraine in previous studies

Region	Gene name	Type	Migraine TWAS				FOCUS	
			Model	R^2^	effect_size	*P* value	PIP	TWAS-Z
1p13.2	*NGF-AS1*	protein	Artery_Aorta	0.081728	-0.181164	2.86E-10	0.998	-6.09
1p36.21	*FHAD1*	protein	Anterior_cingulate_cortex_BA24	0.09331	-0.3653	3.39E-08	0.974	-5.04
			Caudate_basal_ganglia	0.08647	-0.153907	1.93E-07	0.971	-5.01
			Nucleus_accumbens_basal_ganglia_accumbens_basal_ganglia	0.098172	-0.114123	1.66E-07	0.984	-5.12
			Putamen_basal_ganglia_basal_ganglia	0.055866	-0.20457	3.31E-07	0.941	-4.84
2q37.1	*HJURP*	protein	Caudate_basal_ganglia	0.070146	0.587219	2.72E-07	0.992	5.3
			Nucleus_accumbens_basal_ganglia_accumbens_basal_ganglia	0.066215	0.554813	3.42E-08	1	5.92
5q13.3	*POC5*	protein	Amygdala	0.047456	0.164059	2.52E-07	-	1.01
			Anterior_cingulate_cortex_BA24	0.042806	0.090769	2.65E-07	0.484	3.05
			Cerebellar_Hemispher	0.282793	0.0786	1.56E-10	1	6.55
			Cerebellum	0.263836	0.089549	1.72E-07	-	3.22
			Cortex	0.156424	0.123317	2.23E-10	-	1.06
			Frontal_Cortex_BA9	0.05286	0.130486	3.14E-08	-	0.935
			Hippocampus	0.024359	0.282175	1.17E-08	-	-
			Hypothalamus	0.035169	0.119661	4.11E-08	-	-
			Nucleus_accumbens_basal_ganglia_accumbens_basal_ganglia	0.062625	0.125138	2.18E-08	-	0.255
			Spinal_cord_cervical_c-1	0.048626	0.158599	1.17E-08	-	-
5q13.3	*AC010245.2*	lncRNA	Anterior_cingulate_cortex_BA24	0.05817	-0.337346	3.98E-10	-	-4.87
			Caudate_basal_ganglia	0.028894	-0.52382	4.68E-11	0.999	-5.69
			Frontal_Cortex_BA9	0.02653	-0.170674	3.66E-09	-	-
			Hypothalamus	0.121593	-0.225619	6.60E-09	-	-
			Putamen_basal_ganglia_basal_ganglia	0.022971	-0.323605	7.93E-10	-	-
			Artery_Coronary	0.028216	-0.429415	1.21E-06	-	-
7p14.1	*LINC01449*	lncRNA	Artery_Aorta	0.120487	-0.13169	7.01E-15	-	-
			Artery_Coronary	0.025357	-0.665014	3.76E-21	1	-9.05
			Artery_Tibial	0.025121	-0.600408	2.15E-15	-	-
10q23.33	*PLCE1-AS1*	protein	Artery_Tibial	0.052699	-0.125025	1.75E-07	0.9	-4.86
10q23.33	*TBC1D12*	protein	Spinal_cord_cervical_c-1	0.065751	0.26911	3.45E-08	-	-
12p13.32	*CCND2*	protein	Artery_Tibial	0.037212	0.160604	7.97E-10	1	6.45
19q13.2	*B9D2*	protein	Anterior_cingulate_cortex_BA24	0.089763	-0.189229	4.54E-09	0.971	-5.05
			Caudate_basal_ganglia	0.034965	-0.182969	8.13E-08	-	-
			Cerebellum	0.035837	-0.218353	3.20E-08	0.971	-5.39
			Cortex	0.026928	-0.402582	9.32E-11	1	-6.24
			Frontal_Cortex_BA9	0.025896	-0.391737	8.97E-11	1	-6.24
			Hippocampus	0.046359	-0.194721	6.01E-08	-	-
			Hypothalamus	0.099981	-0.277185	3.47E-10	1	-5.84
			Putamen_basal_ganglia_basal_ganglia	0.045991	-0.189611	2.24E-08	-	-
			Artery_Tibial	0.025688	-0.322809	1.02E-10	1	-6.14

### Gene set enrichment analysis based on TWAS results

**Fig. 3:**
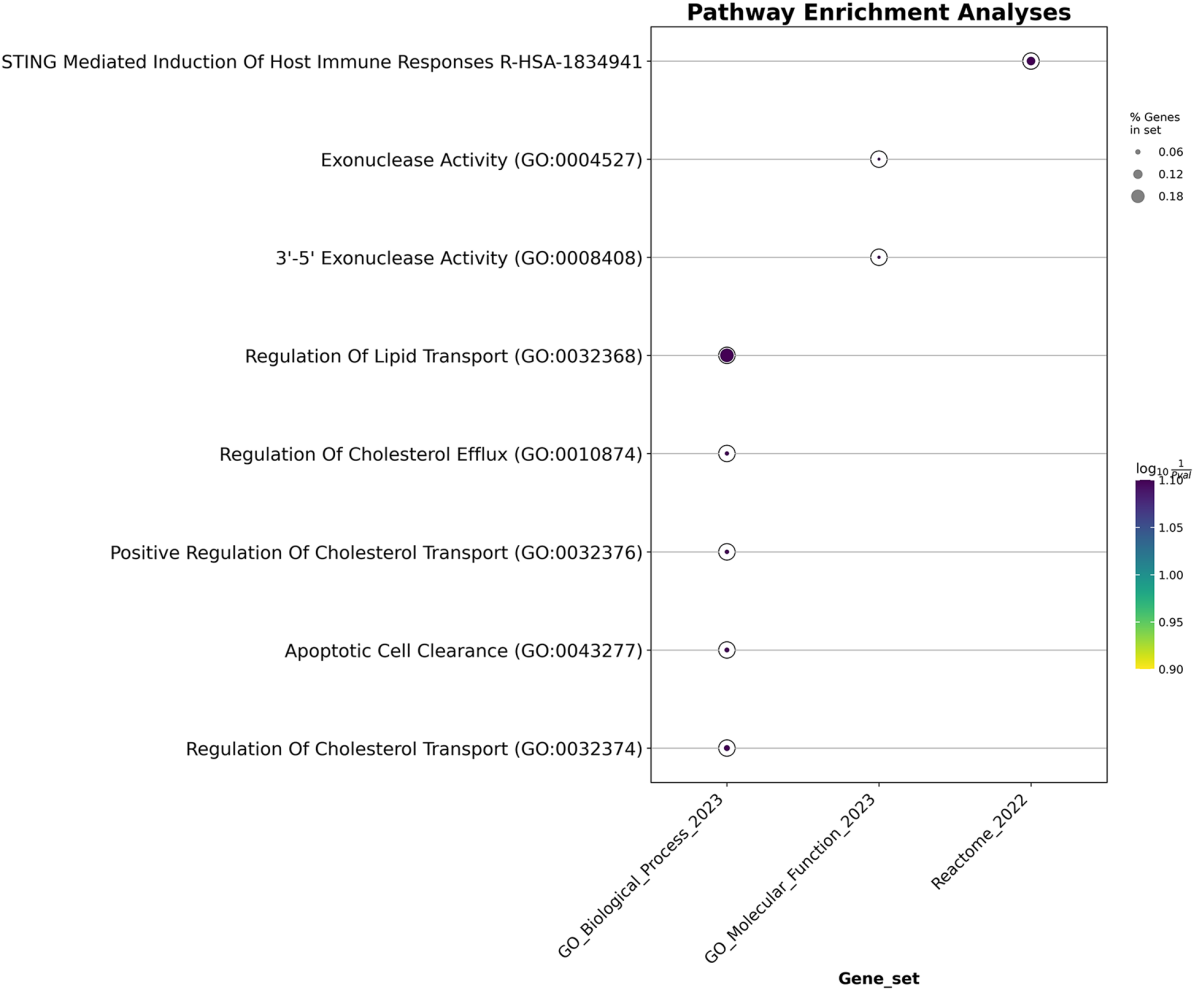
GO-KEGG-Reactome pathway enrichment analyses. **A** Pathway enrichment analysis results of genes identified by TWAS analysis in CNS tissues, exhibiting negative Z-score

Among the risk genes identified in CNS tissues, only the Fanconi anemia pathway was found to be significant. In the enrichment analysis of pathways associated with protective genes, we observed a significant enrichment in pathways pertinent to lipid and cholesterol transport and regulation, nuclease activity, STING-mediated immune responses, and cell apoptosis were significantly enriched (Fig. 3). However, no significant findings were observed in the pathway enrichment results of both risk and protective genes in whole blood and vascular tissues. (Additional file 1: S13-16; Additional file 2: Fig S3).

### Specific cell type annotation

We used the 95 significant genes from the TWAS results as input for the Co-Exp Web analysis. This analysis assigned weight values to the input gene set and enriched them into corresponding modules. We focused on three identified genes, *ICA1L*, *STAT6*, and *UFL1*, which were shared by both PWAS and TWAS analyses, and examined their specific cell type enrichment in different brain regions. The TWAS results for *ICA1L* in whole blood and vascular tissues were significant, but the specific cell types in which it was expressed in vascular tissue were unclear. In the CNS, *ICA1L* was enriched in the Ependymal-External module (*p*-value = 1.128e-07) and the Neuron Interneuron-External module (*p*-value = 0.002184) in the shell nucleus, with a module membership (MM) value of 0.8662 for the latter module. *ICA1L* was also enriched in the amygdala module of the brain, with an MM value of 0.8282. In this module, the meaningful cell types included cortical neurons (*p*-value = 1.238e-44) and cerebral neurons (*p*-value = 2.2e-09). In the Co-Exp analysis, *STAT6* had an MM value of 0.8343 in the spinal cord module and was specifically expressed in microglia (*p*-value 1.661e-69). It also had higher specificity in microglia in the brain hypothalamus module (*p*-value 8.64e-120). The gene *UFL1* was more clearly clustered in the hippocampal and cerebrospinal modules of the brain, with MM values of 0.9178 and 0.9092, respectively. In the hippocampus, *UFL1* was specifically expressed in oligodendrocytes (*p*-value = 0.02051), while in the cerebrospinal cord, it was mainly expressed in cortical neurons (*p*-value = 3.457e-07). The specific cell types for the remaining genes can be found in the supplementary material (Additional file 1: Table S17 and Additional file 2: Table S2).

## Discussion

Migraine is a complex neurological disorder, with both CNS and vascular mechanisms playing significant roles in its pathophysiology [25]. Despite the widespread prevalence of migraine, the contemporary diagnostic and therapeutic approaches warrant further refinement and advancement. In this study, we conducted an integrative analysis of PWAS, TWAS, and FOCUS using data derived from the brain, vascular tissues, and plasma.

We identified eight candidate risk genes for migraine in the ROS/MAP and Banner datasets, and five candidate risk genes for migraine in the plasma pQTL through PWAS. However, we did not observe a clear overlap between the significant proteins identified by brain-based pQTL and those identified by plasma-based pQTL. This lack of overlap could be attributed to several factors. Firstly, the distinct biological environments of the brain and plasma could contribute to this observation. Proteins that play a significant role in the cellular environment of the brain may not have the same prominence in plasma, and vice versa. This discrepancy could be due to differences in protein expression, secretion, degradation, or function between the two tissues. Secondly, the limited power of pQTL, due to the relatively smaller sample size, could have also contributed to this lack of overlap. This limitation underscores the need for further studies with larger sample sizes and more comprehensive proteomic data to provide clearer insights into these observations. Despite the lack of a clear trend, we believe that our findings still provide valuable insights into the potential risk genes for migraine. Each set of proteins identified could be contributing to different aspects of the disease mechanism, reflecting the complex and multifactorial nature of migraine. Additionally, after Bonferroni multiple testing correction, we found 95 risk genes significantly associated with migraine. According to FOCUS analysis, 23 of these genes have a strong causal association with migraine within a 90% confidence interval. Through our analysis of two different brain proteomes using PWAS and TWAS of brain and vascular transcriptomes, we identified three potential causal genes for migraine (*STAT6*, *ICA1L*, and *TREX1)*. However, in our TWAS analysis, regarding genes (*CACNA1A*, *ATP1A2*, and *SCN1A*) associated with monogenic forms of complex migraine, such as Familial Hemiplegic Migraine (FHM), we did not find overlap gene with our identified risk genes [18]. We identified five genes, including *ICA1L*, *TREX1*, *STAT6*, *UFL1*, and *B3GNT8*, which were revealed by both PWAS and TWAS. This shows that the results of this study are consistent at the level of translation and transcription. While, four of these (*ICA1L*, *TREX1*, *STAT6*, and *UFL1*) have been previously reported in association with migraine [26–29]. The discovery of new gene (*B3GNT8*) demonstrates the feasibility of the PWAS study methodology and also benefits from the more novel transcriptomic data we used.

Interestingly, *ICA1L*, *STAT6*, and *UFL1* were further supported by FOCUS (with pip = 0.954,1, and 1, respectively). We also observed that the expression of *STAT6* in whole blood is significantly associated with an increased risk of migraine, while its expression in CNS and vascular tissues is significantly associated with a decreased risk of migraine. This suggests that the effect of *STAT6* on the risk of developing migraine may be tissue-specific. These results suggest that the identified genes may play a role in the regulation of the pathogenesis of migraine and may be potential targets for further research. Our findings underscore the complexity of the genetic basis of migraine and highlight the potential of integrative bioinformatics methods in revealing this complexity.

*ICA1L*, a gene implicated in neuronal signaling, exhibits enriched expression in ependymal cells of the putamen and neurons of the cerebral cortex. This enrichment suggests a potential role for *ICA1L* in the transmission of information within the trigeminal vasculature. Previous research has established a correlation between elevated *ICA1L* expression and a decreased risk of Alzheimer's disease, stroke, and small vessel strokes [30–32]. Furthermore, *ICA1L* has been identified as a shared risk gene between migraine and coronary artery disease (CAD) [26]. The pathophysiological mechanisms of migraine, Alzheimer's disease (AD), and small vessel disease (SVD) may all contribute to the development of white matter damage and cognitive deficits. Vascular dysfunction represents a shared mechanism among these diseases, particularly evident in the context of migraine. Moreover, neuroinflammation, a common factor in the development of both AD and migraine, underscores the potential overlapping pathophysiology among these conditions. Therefore, it can be postulated that *ICA1L* plays a convergent role in the initiation and progression of migraine, AD, and SVD. This shared genetic influence underscores the interconnected nature of these seemingly disparate conditions and highlights the need for further exploration into the multifaceted roles of genes like *ICA1L*. This implies that treatments for these diseases might also aid in migraine management. *UFL1*, a protein-encoding gene, regulates humoral immune processes and endoplasmic reticulum stress, potentially altering vascular morphology and inflammation [25, 33]. Earlier research suggests that *UFL1* plays a role in histone H4 acylation and ATM activation [34]. Our cell-specific analysis shows *UFL1* enrichment in oligodendrocytes and neurons, involved in neuroexcitatory signal regulation and protein modifications in migraine [35, 36]. This suggests *UFL1* as a potential target gene for antihistamines targeting the H4 receptor for migraine prevention and treatment. Evidence suggests that both immune responses and neuroinflammation, observable in peripheral blood, contribute to the pathogenesis of migraine [36, 37]. The *STAT6* gene, in particular, may play a pivotal role in this context. It is hypothesized that *STAT6* may contribute to the activation of the trigeminal vascular system, a process that can trigger an inflammatory response and sustain the state of migraine. This inflammation, potentially manifesting in plasma, could lower the thresholds of injury receptors, leading to heightened sensitization in both central and peripheral regions [25]. Further supporting this hypothesis, *STAT6* shows enriched expression in microglia and macrophages, which are immune cells present in the central nervous system (CNS) and arterial tissues [38, 39]. The activation of these cells can lead to an inflammatory response, thereby potentially exacerbating migraine [40]. *TREX1,* a gene encoding a DNA exonuclease. *TREX1*-deficient brain cells exhibit neuroinflammatory and neurotoxic effects, a critical factor in the pathophysiology of migraine. This signaling pathway contributes to inflammatory responses and the persistence of headaches, key characteristics of migraine [41, 42]. Therefore, *TREX1* may play a significant role in the initiation and maintenance of migraine episodes, potentially through the modulation of neuroinflammatory processes. *B3GNT8* is highly enriched in the esophagus and vagina and is associated with gastrointestinal symptoms such as nausea and vomiting in migraine patients [43]. Given the higher prevalence of migraines in women and the influence of estrogen levels on migraine incidence [44], *B3GNT8* emerges as a key candidate gene for the regulation of migraine-associated gastrointestinal symptoms.. *B3GNT8* is a key candidate gene for regulating migraine-associated gastrointestinal symptoms and hormonal modulation for migraine prevention.

Our research reveals that identified genes play a complex role in migraine development, impacting lipid homeostasis, immune response, cell clearance, and nucleotide metabolism. Enriched pathways related to lipid transport and regulation, particularly cholesterol transport and efflux, suggest a key role for lipid balance in migraine development. This aligns with recent research linking lipid metabolism to migraines, indicating potential for lipid-lowering treatments [45]. We observed enrichment in the STING-mediated immune response pathway, suggesting the genes could regulate immune responses, potentially controlling inflammation-related migraine symptoms. The apoptotic cell clearance pathway was also enriched, indicating a role for the genes in preventing secondary necrosis and inflammation, potentially alleviating migraine symptoms. Finally, enrichment in pathways related to 3'-5' exonuclease activity and nucleobase-containing compound catabolic process suggests involvement in DNA repair and nucleotide metabolism, crucial for genomic stability and cellular balance, disruptions of which could contribute to migraines.

The datasets used in this study encompass a diverse range of proteins, which are crucial for understanding the potential biological mechanisms underlying migraine. These proteins were linked to genetic variation through pQTL analysis, which investigates the influence of genetic variants on protein abundance, measured in the dorsolateral prefrontal cortex (dPFC) region of the brain. However, our study has several limitations. First, the sample size of the migraine GWAS dataset and pQTL data was limited, which may have affected the robustness of our findings. As more migraine GWAS and pQTL data become available in the future, we anticipate that the power and significance of PWAS in understanding diseases like migraine will become more evident. Second, our study was limited by its focus on European populations, which may have influenced the detection of some gene transcriptomic and proteomic expression effects. This limits the generalizability of our findings, and further studies with more diverse populations are needed to validate our results. Third, we only analyzed 17 tissues deemed relevant to migraine, potentially overlooking associations with migraine in other tissues. This includes the possibility that some transcripts are expressed in the brain but not in the blood. Finally, the clinical relevance of our findings requires further validation. The lack of clinical data to correlate with our molecular findings is a significant limitation of this study. Future research should aim to explain how these genes modulate and influence the pathophysiological processes of migraine through scientific experiments. Despite these limitations, by combining these datasets, we were able to identify multiple proteins potentially involved in the development of migraine and gain insights into their potential mechanisms of action.

## Conclusions

In conclusion, by integrating proteomic and transcriptomic data from PWAS and TWAS, we have identified causal genes for migraine, including some that have not been reported in previous TWAS analyses, providing new insights compared to previous TWAS analyses. Our findings shed light on the transcriptomic changes and potential pathogenic mechanisms of these genes in the context of migraine. This makes them promising candidates for future studies aimed at understanding the pathogenesis of migraine and developing effective treatments for this debilitating condition.

### Supplementary Information

**Additional file 1.** The data information used in this study and the analysis files generated, with the title of each table displayed in Additional file1: List.

**Additional file 2:** **Figure S1.** Venn plots of the migraine significant genes. **Figure S2.** FOCUS plot for each gene in one region. **Figure S3.** Pathway network for the migraine significant genes with negative Z-score for 13 central nervous systems tissues (TWAS). **Table S1.** Overlap genes of migraine risk genes identified by TWAS. **Table S2.** Cell type annotation of three important genes.

## Data Availability

The Migraine GWAS dataset provided by Hautakangas et al. can be obtained by contacting the International Headache Genetics Consortium (IHGC) [11]. The interpolation model for the JTI reference transcriptome used in TWAS can be downloaded at https://zenodo.org/record/3842289[8]. The ROSMAP and Banner datasets, including weights and pQTL data, supporting the results of this study can be accessed at https://www.synapse.org/#!Synapse:syn23627957 and the plasma dataset can be downloaded from http://nilanjanchatterjeelab.org/pwas/[12–15]. These resources can be used to further investigate the mechanisms behind GWAS-identified risk variants for migraine. This study used R version 4.2.1 for data collation and analysis. The S-PrediXcan package (https://github.com/hakyimlab/MetaXcan) was used to analyze TWAS [19, 20], while TWAS fine mapping was conducted using the FOCUS package (https://github.com/bogdanlab/focus/) [9, 10]. Gene enrichment analysis was performed with the Enrichr tool (https://maayanlab.cloud/Enrichr/) [21]. The pathway network was built with https://metascape.org/ [22]. The FUSION package was utilized for proteome-wide association studies (PWAS), and cell type-specific analyses were performed using the CoExpNets online tool (https://rytenlab.com/coexp/Run) [23].

## Abbreviations

SNP: Single nucleotide polymorphism
GWAS: Genome-wide association study
PWAS: Proteome-wide association study
TWAS: Transcriptome-wide association study
JTI: Joint-tissue imputation
PIP: Posterior inclusion probability
CNS: Central nervous system
GO: Gene Ontology
KEGG: Kyoto Encyclopedia of Genes and Genomes
eQTL: Expression quantitative trait loci
pQTL: Protein quantitative trait loci
MM: Module membership
