# Supplementary material for: Identifying causal genes for migraine by integrating the proteome and transcriptome
Source: J Headache Pain. 2023 Aug 17;24(1):111. doi: 10.1186/s10194-023-01649-3 (PMC10433568; doi:10.1186/s10194-023-01649-3)
Supplement: Supplementary file 2 — Additional file 2: Figure S1. Venn plots of the migraine significant genes. Figure S2. FOCUS plot for each gene in one region. Figure S3. Pathway network for the migraine significant genes with negative Z-score for 13 central nervous systems tissues (TWAS). Table S1. Overlap genes of migraine risk genes identified by TWAS. Table S2. Cell type annotation of three important genes. [file 10194_2023_1649_MOESM2_ESM.docx]

**Supplementary material for**

**Identifying causal genes for migraine by integrating the proteome and transcriptome**

Shuang-jie Li, Jing-jing Shi, Cheng-yuan Mao, Chan Zhang, Ya-fang Xu, Yu Fan, Zheng-wei Hu, Wen-kai Yu, Xiao-yan Hao, Meng-jie Li, Jia-di Li, Dong-rui Ma, Meng-nan Guo, Chun-yan Zuo, Yuan-yuan Liang, Yu-ming Xu, MD, PhD, Jun Wu, Shi-lei Sun, Yong-gang Wang, MD, PhD^*^ and Chang-he Shi, MD, PhD^*^

*: Corresponding Author

**Supplementary tables**

Figure S1: Venn plots of the migraine significant genes.

Figure S2: FOCUS plot for each gene in one region.

Figure S3: Pathway network for the migraine significant genes (TWAS).

Table S1: Overlap genes of migraine risk genes identified by TWAS.

Table S2: Cell type annotation of three important genes.

**Figure S1: Venn plots of the migraine significant genes.**

Venn plots of migraine risk genes identified in the central nervous system, the whole blood, and 3 vascular tissues by TWAS (JTI models). The numbers in the overlapping regions represent the number of overlapping genes and the numbers in the respective regions indicate the number of genes other than overlapping genes in the model.


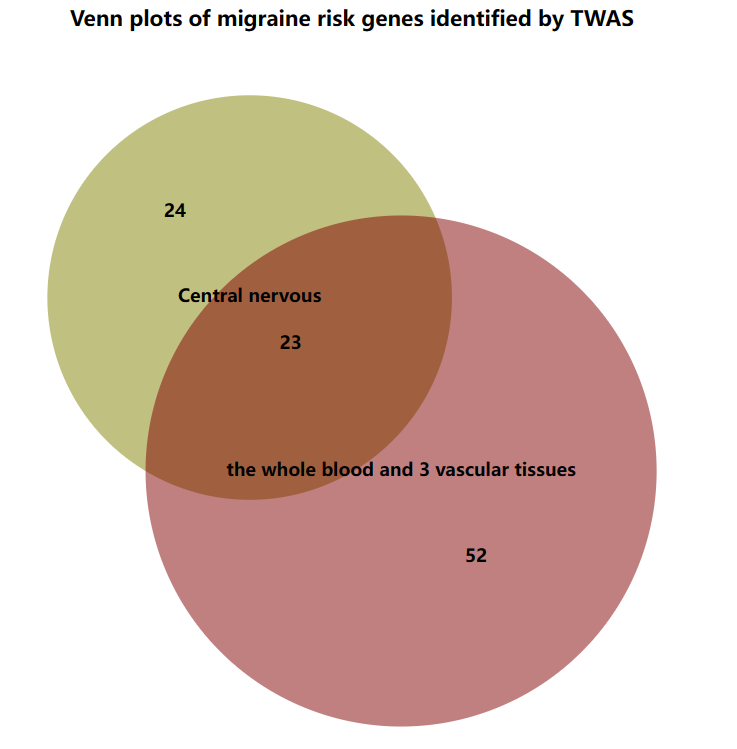


**Figure S2:**

**FOCUS plot for each gene in one region. (A)** The plot contains the predicted expression correlation, TWAS summary statistics, and PIP for each gene in the genomic locus 17: 45876022-17: 47517400 in the brain hippocampus. **(B)** The plot contains the predicted expression correlation, TWAS summary statistics, and PIP for each gene in the genomic locus 10: 104380410-10: 106695048 in the brain cortex. **(C)** The plot contains the predicted expression correlation, TWAS summary statistics, and PIP for each gene in the genomic locus 17: 45876022-17: 47517400 in the whole blood. **(D)** The plot contains the predicted expression correlation, TWAS summary statistics, and PIP for each gene in the genomic locus 17: 45876022-17: 47517400 in the tibial artery.


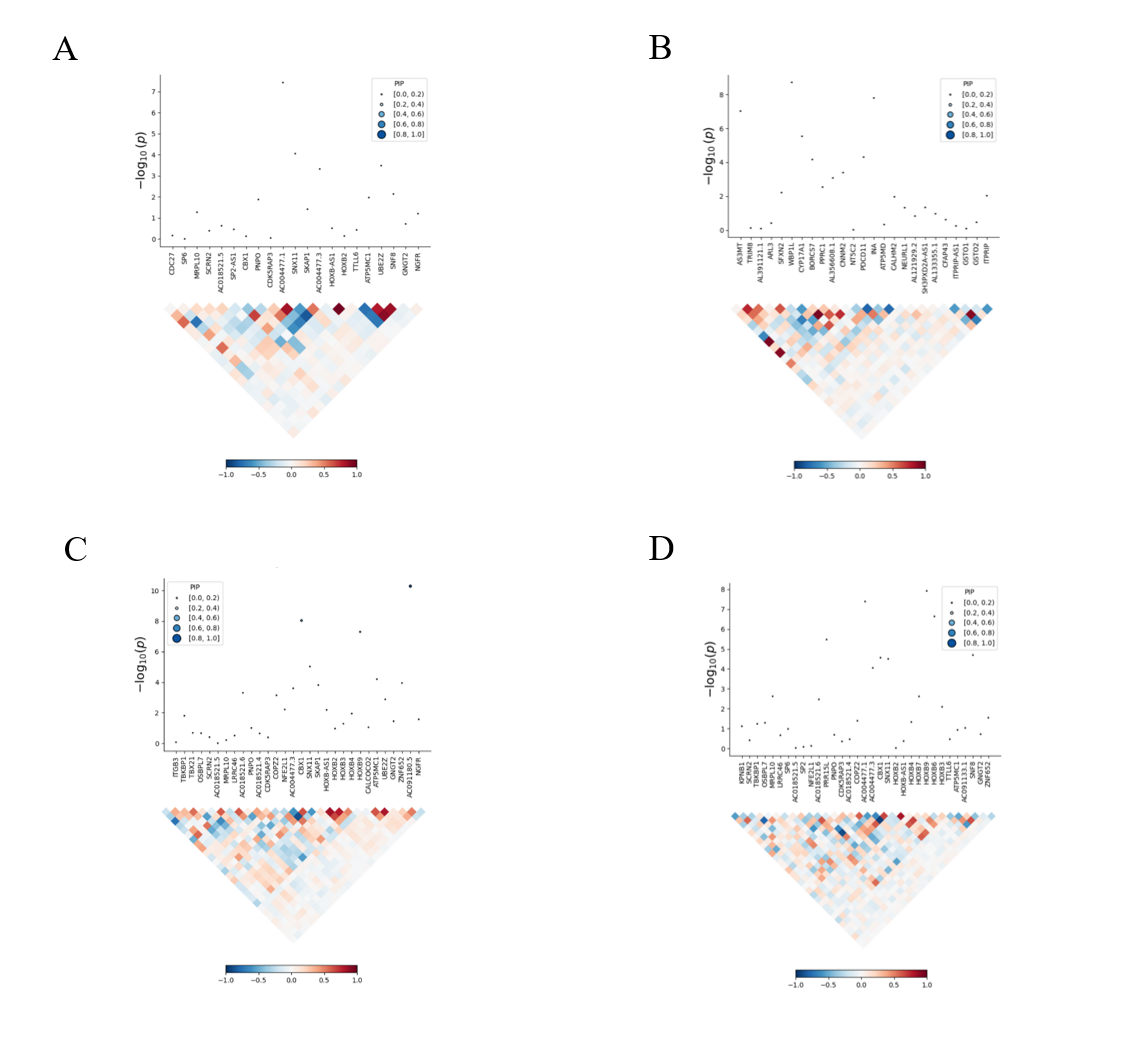


**Figure S3:**

**Pathway network for the migraine significant genes (TWAS).** Pathway network of migraine risk genes with negative Z-score identified in 13 CNS tissues by TWAS (JTI models). The different points of color correspond to different enrichment pathways.

| **Table S1: Overlap genes of migraine risk genes identified by TWAS** | | | | | | | | |
| --- | --- | --- | --- | --- | --- | --- | --- | --- |
| Model | Gene | | | | | | | |
| CNS (24) | *FGF11* | *SLC35G6* | *NXPH4* | *NOL4L* | *NR1H3* | *MACF1* | *FHL3* | *ATRIP* |
|  | *POC5* | *MPPED2* | *MYBPC3* | *POLK* | *AC134772.1* | *WDR12* | *METTL1* | *ZBTB39* |
|  | *CFDP1* | *TBC1D12* | *AL356608.1* | *TMA7* | *AC008026.3* | *FHAD1* | *HJURP* | *LYVE1* |
| Overlap genes (23) | *AC010245.2* | *HPSE2* | *EXOSC5* | *ACTR10* | *REST* | *UFL1* | *TREX1* | *MTF1* |
|  | *MEF2D* | *RAPSN* | *LRP1* | *B9D2* | *PRR13* | *STAT6* | *LINC02641* | *KIAA0040* |
|  | *MDK* | *DMAC2* | *FHL5* | *DGKZ* | *B3GNT8* | *PHACTR1* | *ERICH4* |  |
| the whole blood and 3 vascular tissues (52) | *TSPAN2* | *TEX41* | *CHRM4* | *CCND2* | *FAM117B* | *C12orf4* | *TSPAN12* | *LINC00310* |
|  | *SPAAR* | *PAPPA* | *HRK* | *AL132989.2* | *NGF-AS1* | *IPO8* | *GJA1* | *SLC24A3* |
|  | *MRPS6* | *ICA1L* | *LINC01450* | *IER3* | *KCNK5* | *TJP2* | *NGF* | *HHIPL1* |
|  | *HTRA1* | *HARBI1* | *C1orf87* | *LRP1-AS* | *MRVI1* | *ANKDD1B* | *SHISA5* | *SUGCT* |
|  | *AC073896.4* | *ERC1* | *ADAMTSL4* | *CNNM1* | *BCAR1* | *NT5C2* | *FBXW8* | *PLCE1-AS1* |
|  | *ECM1* | *PLXNB1* | *XCL2* | *C11orf49* | *MRC2* | *TMEM51* |  |  |
|  | *SLC5A3* | *LINC01449* | *ERFL* | *EPHA10* | *CTDSP2* | *PTK2B* |  |  |

| **Table S2: Cell type annotation of three important genes.** | | | |  |  |  |
| --- | --- | --- | --- | --- | --- | --- |
| **Gene** | **Category** | **Network** | **Module** | **Module Membership^a^** | **Module Size^b^** | **Cell Type** |
| *ICA1L* | gtexv6 | Putamen | orangered4 | 0.8662 | 722 | Ependymal-External (p-value 1.128e-07). Neuron_Interneuron-External (p-value 0.002184). Neuron, definite (Cahoy, 2008) (p-value 0.00307). Neuron.Ex1-External (p-value 0.004605). |
| *ICA1L* | gtexv6 | Amygdala | lightgreen | 0.8282 | 875 | Neuron module in Cortex (p-value 1.238e-44). Neuron in Human brain Module (Geschwind,2010) (p-value 2.2e-09). Neuron, pyramidal in network from Sugino/Winden (p-value 2.281e-09). Neuron, probably (Cahoy, 2008) (p-value 0.04429). |
| *STAT6* | gtexv6 | Spinalcord | black | 0.8343 | 438 | Microglia (Type 1) (Geschwind, 2010) (p-value 1.661e-69). Microglia-External (p-value 7.6e-53). Microglia (Type2) module in Cortex (p-value 3.589e-06). |
| *STAT6* | CoExpGTExV7 | BrainHypothalamus | purple | 0.8212 | 645 | Microglia (p-value 8.64e-120). Cortex_MicrogliaDeactivation_Neuroexpresso (p-value 1.574e-70). Cortex_Microglia_Neuroexpresso (p-value 1.4e-63). Hippocampus_MicrogliaDeactivation_Neuroexpresso (p-value 1.631e-55). Cerebellum_MicrogliaDeactivation_Neuroexpresso (p-value 4.923e-54). Hyppocampus_Microglia_Neuroexpresso (p-value 9.25e-51). Cerebellum_Microglia_Neuroexpresso (p-value 7.621e-45). Cortex_MicrogliaActivation_Neuroexpresso (p-value 8.164e-36). Hippocampus_MicrogliaActivation_Neuroexpresso (p-value 2.292e-26). Cerebellum_MicrogliaActivation_Neuroexpresso (p-value 1.559e-21). DAMANIS_MICROGLIA (p-value 3.699e-17). Macrophages (p-value 0.0007083). DARMANIS_FETAL1 (p-value 0.002017). |
| *UFL1* | gtexv6 | Hippocampus | brown4 | 0.9178 | 635 | Oligodendrocytes in Human brain Module (Geschwind,2010) (p-value 0.02051). |
| *UFL1* | gtexv6 | Spinalcord | tan | 0.9092 | 592 | Neuron module in Cortex (p-value 3.457e-07). Neuron in Human brain Module (Geschwind,2010) (p-value 9.001e-07). Neuron, pyramidal in network from Sugino/Winden (p-value 0.02704). |
| a: Represents the degree of membership of the current gene to the current module. When the module membership (mm) is close to 0, the likelihood of that gene belonging to the current module is very low. When the mm is close to 1 or -1, the likelihood of that gene belonging to the current module is very high. | | | | | | |
| b: Total number of genes that have been clustered together within the current module. | | | | | | |
